# Importance of FaWRKY71 in Strawberry (*Fragaria × ananassa*) Fruit Ripening

**DOI:** 10.3390/ijms232012483

**Published:** 2022-10-18

**Authors:** Maolan Yue, Leiyu Jiang, Nating Zhang, Lianxi Zhang, Yongqiang Liu, Yan Wang, Mengyao Li, Yuanxiu Lin, Yunting Zhang, Yong Zhang, Ya Luo, Xiaorong Wang, Qing Chen, Haoru Tang

**Affiliations:** 1 College of Horticulture, Sichuan Agricultural University, Chengdu 611130, China; 2 Institute of Pomology & Olericulture, Sichuan Agricultural University, Chengdu 611130, China

**Keywords:** strawberry, FaWRKY71, abiotic stress, fruit ripening, anthocyanin biosynthesis

## Abstract

WRKY transcription factors play a nonnegligible role in plant growth and development, but little is known about the involvement of WRKY transcription factors in the regulation of fruit ripening. In this study, FaWRKY71 was identified to be closely related to fruit maturation in octoploid strawberry. FaWRKY71 protein localized in the nucleus and responded to cold, salt, low phosphate, ABA, and light quality in strawberry seedlings. The temporal and spatial pattern expression analysis indicated that *FaWRKY71* was expressed in all the detected tissues, especially in the full red fruits. In addition, *FaWRKY71* gave rise to the accumulation of anthocyanin content by promoting the expression of structural genes *FaF3’H*, *FaLAR*, *FaANR*, and transport factors *FaTT19* and *FaTT12* in the flavonoid pathway, and softening the texture of strawberry via up-regulating the abundance of *FaPG19* and *FaPG21*. Furthermore, FaWRKY71 was a positive regulator that mediated resistance against reactive oxygen species by enhancing the enzyme activities of SOD, POD, and CAT, reducing the amount of MDA. Altogether, this study provides new and comprehensive insight into the regulatory mechanisms facilitating fruit ripening in strawberry.

## 1. Introduction

WRKY transcription factors (TFs) are abundant in plants, named for their N-terminus containing highly conserved WRKYGQK amino acid sequence and C-terminus zinc finger motif [1]. According to the number of WRKY signature and zinc finger structures, WRKY TFs are divided into three groups: Group I contains two WRKY domains and C_2_H_2_ zinc finger motif; Group II with one WRKY domain and C_2_H_2_ zinc finger motif and are further divided into five subclasses including IIa, IIb, IIc, IId, and IIe based on the primary amino acid sequence. Group III contains a WRKY domain and C_2_HC zinc finger motif [1,2]. WRKY TFs, binding specifically to W-box [(T) (T) TGAC (C/T)] or W-box like [TGAC (C/T)] cis-regulatory elements [3], assist plants in responding to various stresses.

The first amplified WRKY gene was *SWEET POTATO FACTORS 1* (*SPF1*) from sweet potato [4], since then WRKY TFs have been extensively studied in numerous species. To date, 74, 197, 97, 79, and 127 WRKY TFs have been identified from *Arabidopsis thaliana*, *Glycine max*, *Oryza sativa ssp*. *japonica, grape*, and *Malus* × *domestica*, respectively [2,5,6]. WRKY TFs have either an activating or inhibiting effect on target genes to regulate various physiological processes in plants. They are involved in response to hormonal, pathogens, senescence, wound, and other abiotic stress, such as heat, cold, and salt ions [7]. For instance, *GmWRKY54* activates an abscisic acid (ABA) receptor and an SnRK2 kinase to confer drought tolerance of soybean [8]. *AhWRKY75* endows transgenic peanuts with salt tolerance by accelerating the efficiency of reactive oxygen species scavenging (ROS) system [9]. In banana fruit, *MaWRKY31*, *MaWRKY33*, *MaWRKY60*, and *MaWRKY71* directly activate ABA biosynthesis-related gene *NECD* expression and improve ABA level, resulting in ABA-induced cold tolerance [10]. *FaWRKY11* raises the level of disease-resistance genes to delay the occurrence of *Botrytis cinerea* in strawberry fruit [11]. *FvWRKY71* confers early flowering via exerting an activation effects on flowering-related genes *FvFUL*, *FvSEP1*, *FvAGL42*, *FvLFY*, and *FvFPF1* in woodland strawberry [12]. Studies have reported that WRKY TFs can also co-regulate plant secondary metabolism by interacting with other TFs. For example, *MdWRKY40* interacts with *MdMYB1*, a positive regulatory factor of anthocyanin, to induce wounding-mediated anthocyanin accumulation in apple [13]. *PyWRKY26* and *PybHLH3* co-target the *PyMYB114* promoter to regulate anthocyanin accumulation in red-skinned pear [14].

Strawberry (*Fragaria × ananassa* Duch.) is a perennial herb in the Rosaceous family, and is chosed by consumers for its bright color, unique flavor, abundant flavonoid substances, and is widely cultivated worldwide [15]. Strawberry fruit ripening involves a series of physiological changes resulting in modification to fruit shape, size, texture, and pigmentation, which is contributed by gene expression and enzyme activity, and requires a complex set of environmental and endogenous signals, such as light, temperature, and hormones [16]. ABA and auxin (especially IAA) are the main hormones that cooperatively regulate the development of some non-climacteric fruits like strawberry [17]. As the fruit ripens, the initiation of an increase in ABA levels coincides with a decrease in IAA levels [18], ABA promotes the transport of IAA from achene to receptacle while inhibiting the biosynthesis of IAA in receptacle [19]. The decrease in ABA content is attributable to the application of exogenous auxin due to a reduced expression level of *9- cis –epoxycarotenoid dioxygenase* (*FaNCED*), a key ABA rate-limiting enzyme. Besides, the increased expression abundance of ripening related genes, such as *phenylalanine ammonia lyase* (*PAL*) which is involved in anthocyanin biosynthesis could be induced by ABA [20]. Strawberry fruit softens dramatically at the last stages of maturation, during this process a variety of enzymes and proteins act cooperatively on different enzyme cell wall polysaccharides. It has commonly been assumed that *polyga lacturonase* (*PG*) and *pectate lyase* (*PL*) are directly related to strawberry fruit softening [21]. Transgenic silenced lines of *FaPG1* showed a significant role for this in fruit firmness [22]. The level of *PG* transcript in the softer strawberry cultivar ‘Toyonoka’ increases significantly at the late fruit ripening stage, whereas a lowerlevel was detected in the harder cultivar ‘Sweet Charlie’ [23]. Previously published studies on the effect of WRKY TFs are focused on resilience to biotic or abiotic stresses [7,24], which failed to resolve the potential association between WRKY and fruit ripening. The number of WRKY proteins in the ‘Camarosa’ and ‘Reikou’ genomes of cultivated strawberries was 222 and 47, respectively, due to different WRKY domains in the two genomes [25,26].

In this study, using the transcriptome data of cultivated strawberry fruits treated with different light quality [27], we screened out a WRKY TF FaWRKY71, which was found to be differentially expressed in red and blue light. We speculated that its expression might be induced by different light qualities and related to anthocyanin accumulation in strawberry fruit. On this basis, the CDS and promoter sequences of *FaWRKY71* were cloned from the cultivated strawberry ‘Benihoppe’, and the protein localization, tissue specificity, and response patterns under different light qualities, low temperature, drought, ABA, and salt stress were studied. Furthermore, the transient overexpression of *FaWRKY71* in strawberry fruits was performed. Our study revealed the response of *FaWRKY71* to strawberry abiotic stress and explored the molecular mechanism of *FaWRKY71* affecting fruit ripening. These findings will enhance our ability to regulate fruit ripening and obtain longer shelf life.

## 2. Results

### 2.1. Sequence Analysis and Subcellular Localization of FaWRKY71

**Figure 1:**
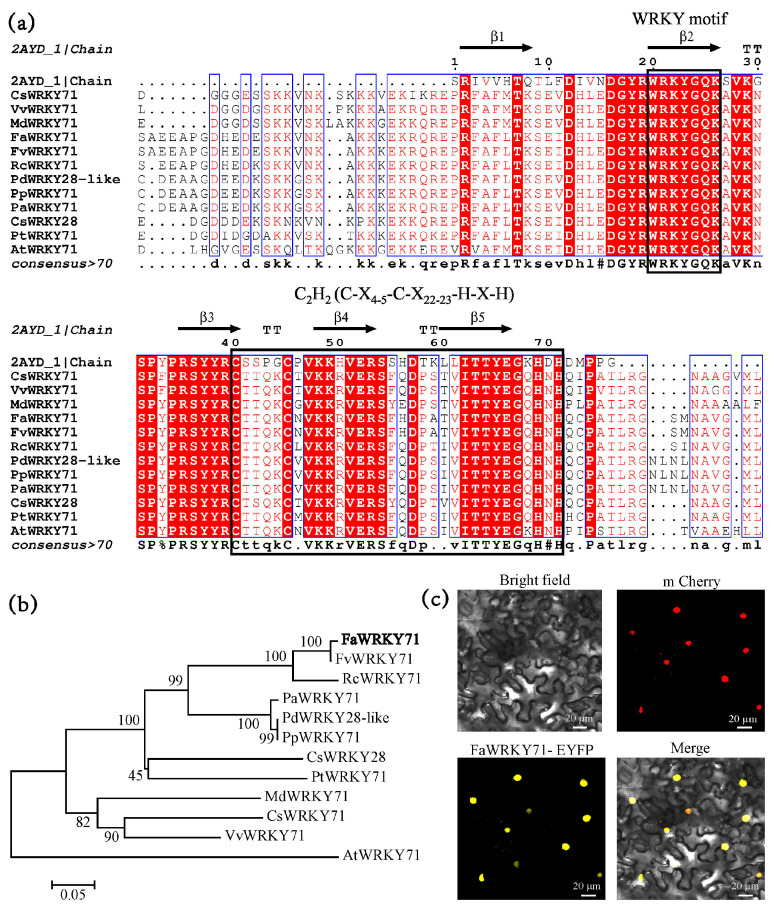
Sequence analysis and subcellular localization of FaWRKY71. (**a**) Alignment of the WRKY motif and C2H2 zinc finger structure of FaWRKY71 proteins with it′ s homologous in other species. The black box indicates the WRKY domain and C2H2 motif. (**b**) Phylogenetic analysis of FaWRKY71. The bold font stands for FaWRKY71. FvWRKY71 (*Fragaria vesca*): XP_004303874.1; RcWRKY71 (*Rosa chinensis*): XP_024182610.1; PaWRKY71 (*Prunus avium*): XP_021811687.1; PdWRKY28-like (*Prunus dulcis*): XP_034208919.1; PpWRKY71 *(Prunus persica*): XP_007217821.1; CsWRKY28 (*Citrus sinensis*): KAH9770763.1; PtWRKY71 (*Populus trichocarpa*): XP_002306743.1; MdWRKY71 (*Malus domestica*): XP_008383508.2; CsWRKY71 (*Camellia sinensis*): XP_028100462.1; VvWRKY71 (*Vitis vinifera*): XP_002272089.1; AtWRKY71 (*Arabidopsis thaliana*): NP_174279.1. (**c**) Subcellular localization of FaWRKY71 protein. Scale bars represent 20 µm.

The complete coding sequence (CDS) of *FaWRKY71* was 1,119 bp, encoding a polypeptide consisting of 372 amino acid residues with a predicted molecular mass of 42.1 kDa, and a theoretical pI of 6.43. The grand average of hydropathicity was −0.991, the protein was presumed to be hydrophilic. HMMER and SignalP analysis showed that FaWRKY71 had no transmembrane structure or signal peptide, which was a non-secretory protein. FaWRKY71 belonged to Group II for it contained a WRKY motif and the zinc-finger structure was C_2_H_2_ (C-X_4-5_-C-X_22–23_-H-X-H) (Figure 1a). To analyze the evolutionary relationship between FaWRKY71 and other WRKY71 proteins, the deduced amino acid sequences of FaWRKY71 and its homologous genes were clustered using the Neighbor-Joining method. The proteins were clustered into two clades (Figure 1b), AtWRKY71 was grouped into one clade, and the rest of them were classified into another. FaWRKY71 was most similar to FvWRKY71, and clustered into the same clade with RcWRKY71, indicating their functional similarity. The subcellular localization results showed that the FaWRKY71-EYFP fusion protein was localized in the nucleus (Figure 1c), and it was speculated that FaWRKY71 may be involved in the regulation of gene expression.

### 2.2. Analysis of FaWRKY71 Promoter

The *FaWRKY71* promoter (accession: OP243273) was amplified with a length of 1644 bp and the cis-elements analyzed using the Plant CARE online website. It contained a large number of light-responsive elements (G-Box, AE-box, Box 4, ATCT-motif, MRE, and sp1), MYB binding site, and hormone-responsive elements, such as ABA-responsive element ABRE, auxin-responsive element TGA-box and MeJA-responsiveness element CGTCA-motif and so forth (Table 1). Indicating that FaWRKY71 was likely to be induced by abiotic stresses.

### 2.3. Expression Patterns of FaWRKY71 in Different Tissues under Multiple Abiotic Stresses

**Figure 2:**
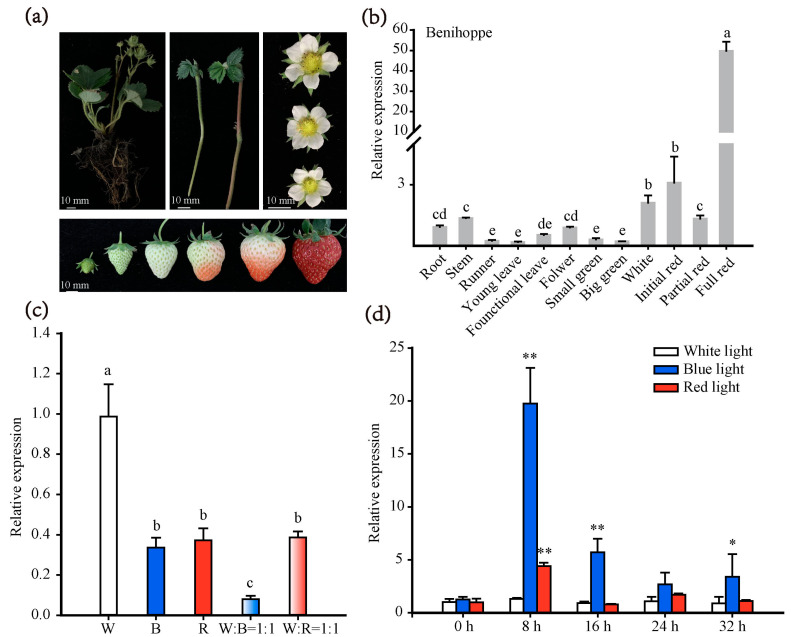
Expression characteristics of *FaWRKY71*. (**a**) Diagrams about different tissues of ‘Benihoppe’. Scale bars represent 10 mm. (**b**) Spatial and temporal expression of *FaWRKY71* in different tissues and different developmental stages of strawberries. Multiple comparisons were tested using Turkey’s test and significant differences (*p* < 0.05) were indicated by different lowercase letters. (**c**) Effects of different light qualities on *FaWRKY71* gene expression in strawberry full red fruits. W (White light); B (Blue light); W: B = 1:1 (White light: Blue light = 1:1); W: R = 1:1 (White light: Blue light = 1:1). (**d**) Effects of different light qualities on *FaWRKY71* gene expression in tissue culture seedlings of strawberry. Error bars were SEs for three replicates, and statistical significance was determined with the white light by Student’s t-test (**, *p* < 0.01; *, *p* < 0.05).

The temporal and spatial pattern expression analysis of *FaWRKY71* from root to fruit was conducted using qRT-PCR. The results showed that *FaWRKY71* was expressed in various tissues of strawberry (‘Benihoppe’), but mainly concentrated in the fruits. The expression level of *FaWRKY71* slowly increased from white to partial red stage and then reached the highest level once ripening had initiated (Figure 2a,b). The results indicated that *FaWRKY71* may be involved in the process related to strawberry ripening.

In view of the fact that the promoter sequence of *FaWRKY71* contained a large number of light-responsive and stress-responsive cis-elements, different light qualities and various abiotic stresses were applied to clarify the inducible characteristics of *FaWRKY71*. In strawberry fruits, both red and blue light significantly reduced the relative expression of *FaWRKY71* compared with the white light. The mixed light treatments also attenuated the expression abundance of *FaWRKY71* and the lowest was in white light: blue light = 1:1 (Figure 2c). These results showed that blue and red light, especially blue light, could inhibit the expression of *FaWRKY71* in fruit. In strawberry seedlings, blue light promoted the expression of *FaWRKY71*, but the growth trend decreased with the prolongation of illumination time and reached the maximum at 8 h (Figure 2d). In comparison, the effect of red light on the expression of *FaWRKY71* was not as marked as that of blue light, which had been raised only at 8 h. These results implied that *FaWRKY71* has different response patterns to red or blue light qualities in strawberry fruits and tissue culture seedlings.

**Figure 3:**
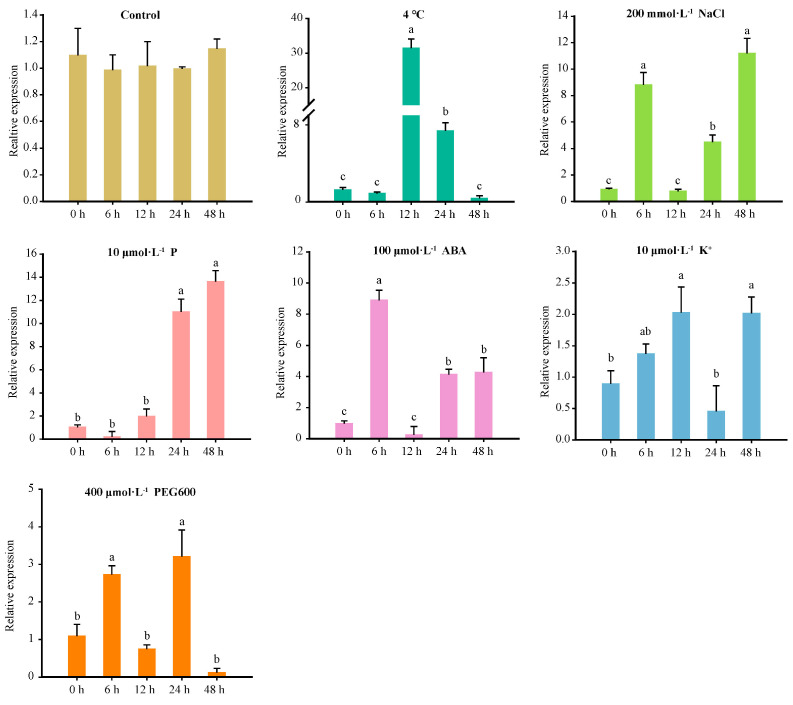
Expression patterns of *FaWRKY71* under different abiotic stresses. Multiple comparisons were tested using Turkey’s test and significant differences (*p* < 0.05) were indicated by different lowercase letters.

The abiotic stress treatments of tissue culture seedlings showed that *FaWRKY71* was significantly induced by cold (4 °C). The relative expression level of *FaWRKY71* peaked at 12 h 4 °C induction and reached up to 30-fold more than that of the control, then reduced to a comparable level with the control at 48 h of induction (Figure 3). In addition, *FaWRKY71* responded to salt, low phosphate, and ABA. There was a relatively similar variation tendency against salt and ABA during the treatment period, which was significantly increased at 6 h, then fell sharply, and finally rose again. This may be due to the expression of *FaWRKY71* altering the tolerance of strawberries against those two kinds of stresses. For phosphate deficiency, *FaWRKY71* was not upregulated until 24 h treatment and maintained the highest level at 48 h. The persistent high expression suggested that it may play a positive regulatory role in the signal transduction pathway of strawberries in response to phosphate deficiency. Nevertheless, low potassium and drought treatment caused a faint increase in the expression level of *FaWRKY71*. Our data, suggested that *FaWRKY71* could respond to the induction of six above abiotic stresses.

### 2.4. Accumulation of Anthocyanin, Lignin and Fruit Ripening

**Figure 4:**
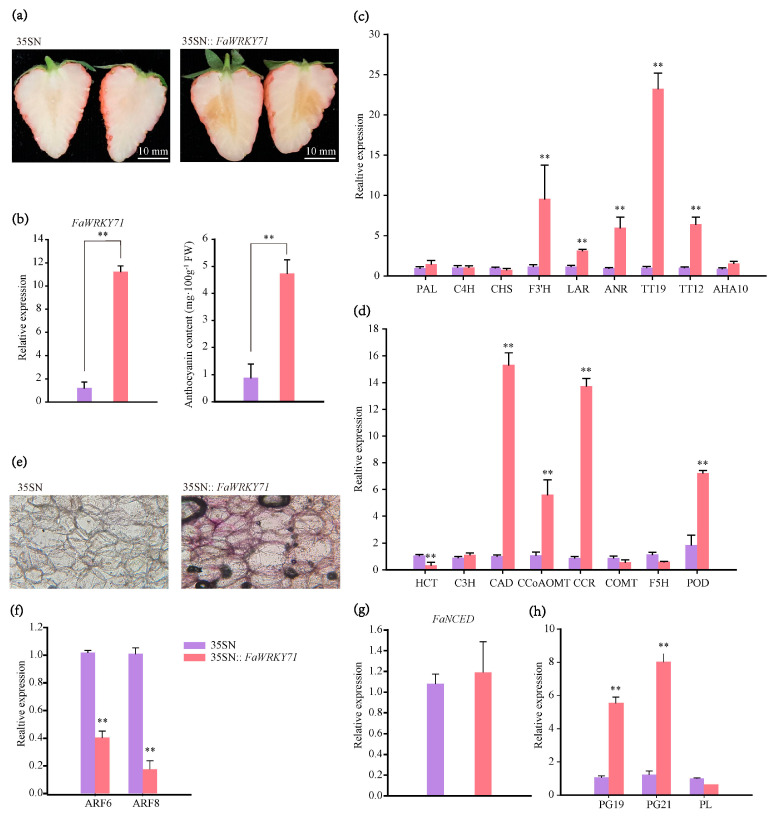
*FaWRKY71* increased the accumulation of anthocyanin and lignin and contributed to fruit softening. (**a**) The phenotype of overexpressed *FaWRKY71* in ‘Xiaobai’ fruit. 35SN (empty vector) was as control. Scale bars represent 10 mm. (**b**) Relative expression level of *FaWRKY71* and anthocyanin content in it′ s overexpressed samples. Error bars were SEs for three replicates, and statistical significance was determined by Student’s t-test (**, *p* < 0.01). (**c**) qRT-PCR analysis of key structural genes and transport factors in the flavonoid biosynthesis pathway. *Phenylalanine ammonia lyase* (*PAL*); *cinnamate 4-hydroxylase* (*C4H*); *chalcone synthase* (*CHS*); *flavonoid 3′-hydroxylase* (*F3′H*); *leucoanthocyanidin reductase* (*LAR*); *anthocyanidin reductase* (*ANR*); *TRANSPARENT TESTA 19* (*TT19*); *TRANSPARENT TESTA 12* (*TT12*); *H^+^-ATPase 10* (*AHA10*). (**d**) qRT-PCR analysis of key structural genes of lignin pathway. *shikimate O-hydroxycinnamoyl transferase* (*HCT*); *coumarate 3-hydroxylase* (*C3H*); *cinnamyl alcohol dehydrogenase* (*CAD*); *caffeoyl CoA O-methyl transferase* (*CCoAOMT*); *cinnamoyl CoA reductase* (*CCR*); *caffeic acid O-methyltransferase* (*COMT*); *ferulate 5-hydroxylase* (*F5H*); *peroxidase* (*POD*). (**e**) Safranin-stained section of strawberry fruit. The lignified cell walls were dyed red. (**f**–**h**) The expression levels of *ARF*, *NCED*, *PG*, and *PL* in *FaWRKY71* overexpressed samples.

To explore whether the augmented level of *FaWRKY71* in strawberry fruit at the color transformation stage is related to anthocyanin accumulation, transient overexpression of *FaWRKY71* was performed in the ‘Xiaobai’ fruits. Compared to the control, a faint and diffuse pigmentation could be observed in *FaWRKY71* overexpressed samples, and the anthocyanin content was significantly higher than that in the control (Figure 4a,b). qRT-PCR analysis showed that the structural genes *FaF3′H*, *FaLAR*, and *FaANR*, as well as some transporters *FaTT19*, and *FaTT12* in the anthocyanin and procyanidin (PA) metabolic pathway were markedly induced (Figure 4c).

Lignin and anthocyanin shared the common phenylpropane metabolic pathway, and lignin, as an important component of the plant cell wall, also played a vital role in resisting stress. The relative expression levels of key structural genes in the lignin metabolic pathway were also examined. The results showed that *FaCAD*, *FaCCoAOMT*, *FaCCR*, and *FaPOD* were all augmented (Figure 4d). Additionally, the strawberry fruit was stained with safranin to test whether the cell wall was lignified. Results demonstrated clearly that hard-lignified cell walls were stained red in the overexpressed samples, but not in the control, despite partial degradation of cell walls due to softening of ripe fruits (Figure 4e). Moreover, cell contours in the overexpressed samples were clearer, indicating that FaWRKY71 stabilized cell wall structure by enhancing the degree of lignification in mature strawberry fruits. Furthermore, the expression of auxin response factors *ARF6* and *ARF8* were diminished, while *NCED* was unchanged (Figure 4f,g). The expression of fruit softening-related enzymes genes *PG19* and *PG21* were enhanced, and *pectate lyase* (*PL*) was not (Figure 4h). These data reported here support the assumption that FaWRKY71 accelerated the ripening process of strawberry fruit by affecting auxin, anthocyanin, and cell wall catabolism-related genes.

### 2.5. Antioxidant Ability of Strawberry Fruit

**Figure 5:**
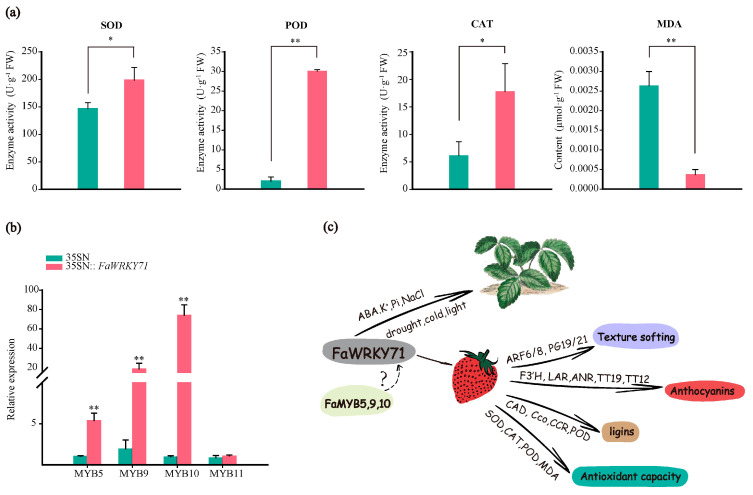
The activity of antioxidant enzymes in *FaWRKY71* overexpressed samples and Regulatory model of FaWRKY71 in strawberry. (**a**) Enzymes activity of SOD, POD, CAT and the content of MDA. Error bars were SEs for three replicates, and statistical significance was determined by Student’s t-test (**, *p* < 0.01; *, *p* < 0.05). (**b**). Relative expression of *MYBs* in *FaWRKY71* overexpressed samples. (**c**) A model indicating how FaWRKY71 regulates strawberry ripening.

To investigate the effect of *FaWRKY71* on the removal of ROS in strawberry fruit, the activity of key antioxidant enzymes including Catalase (CAT), Superoxide dismutase (SOD), and Peroxidase (POD) as well as the content of Malondialdehyde (MDA) were determined. The results showed that the enzyme activities of SOD, POD, and CAT in *FaWRKY71* overexpressed samples were increased substantially, and the content of MDA was greatly reduced (Figure 5a). The data reported here indicated that *FaWRKY71* was of great benefit to improve the scavenging ability of ROS in strawberry fruits, and effectively reducing the content of MDA to limit the damage to strawberry fruits.

## 3. Discussion

*WKRY* genes family is widely represented in higher plants and play an important role in response to various biotic and abiotic stresses. WRKY TFs have been studied in many species. In strawberries, reports on *WRKY* genes have mainly focused on the diploid wild strawberry. In 2019 and 2022 [25,26], Chen and Zou identified the *WRKY* families in the cultivated strawberries ‘Camarosa’ and ‘Reikou’, respectively. Little is known about *WRKY* in relation to ripening in cultivated strawberries. Moreover, studies on the induction characteristics of *FaWRKY71* are still unclear. Here, the tissue-specific manner and induction characteristics of *FaWRKY71* in strawberries under several abiotic stresses were investigated. It had been reported that *FvWRKY71* was highly expressed in the shoot apex and red fruit and led to early flowering in strawberries [12]. In line with previous studies, we found that *FaWRKY71* was highly expressed once fruit ripening had initiated in ‘Benihoppe’, so we hypothesized that *FaWRKY71* was related to fruit ripening. The results of the experiment confirmed that *FaWRKY71* stimulated anthocyanin accumulation by augmenting the relative expression abundance of flavonoid biosynthesis genes *FaF3’H*, *FaLAR*, *FaANR*, and anthocyanin transport genes *FaTT19* and *FaTT12* in strawberry fruit. Our results also demonstrated that *FaWRKY71* promoted fruit ripening by accelerating anthocyanin accumulation.

*FaWRKY71* was responsive to seven abiotic stresses (light, low phosphate, low potassium, cold, ABA, salt, and drought). In the previous analysis of the transcriptome data of ‘Toyonoka’ strawberry fruits treated with red and blue light, we found that *FaWRKY71* was down-regulated in both red and blue light treatment. [27]. Consistent with the previous study, we found blue light, red light, white light: blue light = 1:1, and white light: red light = 1:1 all inhibited the expression of *FaWRKY71* compared to white light treatment in strawberry fruit. For tissue culture seedlings, the relative expression of *FaWRKY71* was significantly up-regulated under blue light treatment for 8 h, 16 h, 32 h and red light treatment for 8 h. Indicating that there are differences in the response patterns of *FaWRKY71* to different light qualities in strawberry fruits and tissue culture seedlings. It has been reported that different light quality had different effects on plant growth and fruit coloring [28,29]. For example, blue and red light could promote anthocyanin accumulation in ‘Toyonoka’ strawberry fruit, but had no obvious effect on ‘Tokun’ fruit [27,30]. Therefore, strawberry genotypes and varieties can also change the effect of light quality on strawberry. Many studies have shown that WRKY conferred cold tolerance in a variety of plants. *KoWRKY40* transgenic Arabidopsis plants exhibited higher SOD, POD, and CAT activities, and lower MDA content under cold stress [31]. *CsWRKY46* from cucumber endowed transgenic plants with cold tolerance and positively regulated cold signaling pathway, specifically interact with the W-box in the promoter of ABA-responsive transcription factor ABI5. [32]. Our study also found that *FaWRKY71* can quickly respond to cold stress, and affect the antioxidant system, but whether this process is mediated by an ABA manner merits further investigation. In Arabidopsis, long-term Pi deficiency elicited anthocyanin accumulation, for flavonol 3-*O*-glucosyltransferase, dihydroflavonol-4-reductase, anthocyanidin synthase, and flavonone-3-hydroxylase in the anthocyanin biosynthetic pathway were induced during Pi deprivation [33]. The suppression of *AtWRKY75* resulted in an early anthocyanin accumulation during Pi starvation, while the anthocyanin content in the RNAi seedlings did not vary significantly under Pi-sufficient conditions [34]. These results implied that low phosphorus did affect anthocyanin biosynthesis. Directly in line with Devaiah’s findings, we found that the expression levels of *FaWRKY71* was enhanced under low Pi stress, which confirmed that *FaWRKY71* could be induced by Pi starvation. Surprisingly, *FaWRKY71* reinforced the abundance of several structural genes in the anthocyanin biosynthesis pathway under Pi-sufficient conditions. Therefore, it is reasonable to speculate that either low phosphorus stress or *FaWRKY71* contribute to anthocyanin accumulation.

Anthocyanin is the main pigment that determines the color of strawberry fruit and is also a sign of fruit ripening. Anthocyanin accumulation is not only affected by external factors such as light, temperature, and exogenous hormones but also the result of the co-expression of structural genes in the anthocyanin biosynthesis pathway, which are comprehensively controlled by MYB, bHLH, and WD40 TFs [35]. An et al. (2019) [13] argued that *MdWRKY40* interacted with *MdMYB1* directly and enhanced anthocyanin accumulation. *McWRKY71* was induced by O_3_ stress and then interacted with *McMYB12* to stimulate PA biosynthesis [36]. The positive MYB regulators of anthocyanin include *MYB5* (unpublished), *MYB9*, *MYB10*, and *MYB11* [37,38,39]. In this paper, except for *FaMYB11*, the relative expression of *FaMYB5*, *9*, and *10* were all raised in *FaWRKY71* overexpressed samples, especially *FaMYB9* and *FaMYB10* (Figure 5b). It can be hypothesized that the positive effect of *FaWRKY71* on strawberry anthocyanin pigmentation is closely related to *FaMYB5*, *9*, or *10*. In addition to anthocyanin accumulation, strawberry fruit ripening is accompanied by texture softening, the ripening-related softening of fleshy fruits is a consequence of the enzyme-mediated cell wall degradation, which leads to the solubilization and depolymerization of cell wall components [40,41,42]. The primary wall is composed of pectin and glycoprotein and the secondary wall is composed of cellulose, hemicellulose, and lignin, which are the main fractions to maintain the cell morphology of strawberry. During fruit ripening and softening, a large amount of pectin is degraded [21]. The findings of this study suggested that the expressions of *FaPG19* and *FaPG21*, which play an important role in fruit ripening and softening, were ascended in *FaWRKY71* overexpressed samples. Signaling that *FaWRKY71* expedites fruit ripening by degrading pectin, and maintains the cell morphology as much as possible by increasing the content of lignin simultaneously.

ABA and auxin synergistically regulate fleshy fruit ripening in non-climacteric fruit like strawberry. Auxin is producted by immature achenes which promotes the division and expansion of receptacle cells [43]. With the development of strawberry fruits, the concentration of auxin declined and accelerates the rate of fruit ripening, indicating that auxin content is negatively correlated with fruit ripening. On the contrary, the level of ABA rises at the white stage, and increases rapidly until the red stage, which is consistent with the onset of fruit coloring and anthocyanin accumulation [17,44]. Xu et al. showed that MiR167 delayed strawberry fruit senescence by targeting *ARF8* under low temperature [45], the *ARF6* and *ARF8* single mutation leading to parthenocarpic fruit [46]. Our results demonstrate that the expression of *FaARF6* and *FaARF8* fell precipitously in *FaWRKY71* overexpressed samples. This means that *FaWRKY71* may arrest the content of auxin by restraining *FaARF6* and *FaARF8*, service to fruit ripening. Previous studies have reported that *MaWRKY71* was induced by exogenous ABA treatment and directly bound to the promoters of ABA biosynthesis-related genes *MaNCED1* and *MaNCED2*, thereby enhancing cold tolerance in banana fruit [10]. Here, we found that *FaWRKY71* was augmented under the induction of exogenous ABA in tissue culture seedlings, indicating that *FaWRKY71* could respond to ABA. Aiming to explore the intrinsic link between *FaWRKY71* and ABA, the key factor *NCED* was detected in *FaWRKY71* overexpressed samples, it was important to note that the expression level of *FaNCED* remained unchanged, we speculated that the expression of *FaWRKY71* was induced by exogenous ABA, whereas *FaWRKY71* did not affect the synthesis of endogenous ABA. It was more noteworthy that *FaWRKY71* promoted strawberry fruit ripening through auxin rather than an ABA manner.

A large amount of ROS will be produced during plant growth. If not removed in time, oxidative damage will be caused at the cellular and molecular levels [47]. Plants have evolved a set of detoxification mechanisms. In addition to dehydrogenation and polymerization of monomers to lignins, POD is also one of the key factors in the enzymatic defense system of plants under adverse conditions, specifically, it removes ROS and improves the stress resistance of plants. POD works in concert with CAT and SOD. According to Zhang (2022) [36], *McWRKY71* promoted the accumulation of anthocyanin and PA, increased the ability to scavenge ROS and resist O_3_ stress in *Malus*, the author held the view that the protection against ozone stress was attributed to the increment of anthocyanin and PA, for they also possess antioxidative activities and scavenge ROS [48]. In the light of WRKY TF being able to activate or inhibit transcription of a physiological process by combining W-box elements on the target gene promoter, in inconsistency with Zhang, we believe that the enhanced antioxidant capacity in strawberry fruits was caused by the superposition of *FaWRKY71* and anthocyanin.

## 4. Materials and Methods

### 4.1. Plant Materials and Treatments

Octoploid strawberry cultivars ‘Benihoppe’ and ‘Xiaobai’ were grown in an environmentally controlled chamber at Sichuan Agricultural University at 24 °C, 5500 lx, and 80% humidity. Fruits at different developmental stages (small green, big green, white, initial red, partial red, and full red) were collected at 7, 15, 22, 28, 36, and 40 days after anthesis. The production seedlings at the white fruit stage with the same growth vigor were treated with different light qualities in a LED incubator. Each plant kept 2 fruits at least and was exposed to white light, red light, blue light, red light: white light = 1:1, and blue light: white light = 1:1 for 10 d until the fruit ripens, and collect the full red fruits. The 45 d tissue culture seedlings were treated with red light (730 nm) and blue light (450 nm) in a red-blue light combined plant growth chamber for light (16 h) – dark (8 h) –light (8 h), and white light was used as the control. Samples were collected at 0, 8, 16, 24, and 32 h, respectively. For abiotic stress treatment, 45 d tissue culture seedlings were inoculated in 1/2 MS basic medium for 1 week, and then subjected to ABA (100 μmol·L^−1^), 4 °C, PEG6000 (400 μmol·L^−1^, simulated drought), NaCl (200 mmol·L^−1^), low P (10 μmol·L^−1^), low K^+^ (10 μmol·L^−1^) treatments. The stems and leaves were collected at 0, 6, 12, 24, and 48 h. All the collected samples were quick-frozen with liquid nitrogen and stored at −80 °C for further analysis. Three biological replicates were set for all the above treatments, and each replicate included 10 production seedlings or tissue culture seedlings.

### 4.2. Amplification of FaWRKY71 and the Promoter of FaWRKY71

Total RNA from samples was extracted using a modified CTAB method [49], and cDNA was generated by the Prime Script™ RT reagent Kit with gDNA Eraser (TaKaRa, Dalian, Chian). Taking the *FaWRKY71* sequence obtained from the previous transcriptome as a reference [27], two specific primers FaWRKY31-F and FaWRKY31-R (Table S1) were designed using Oligo 7.0 software (Version: 7.53, 1267 Vondelpark, Colorado Springs, CO, USA), the PCR product was amplified and sent to Shanghai Sangon for sequencing. The DNA of the ‘Benihoppe’ was extracted followed by Jiang′s method [50]. Two specific primers ProFaWRKY31-F and ProFaWRKY31-R (Table S1)were designed with reference to the genome sequences of diploid strawberry and cultivated strawberry registered on GDR (Genome Database for Rosaceae), PCR product was amplified and sent to Shanghai Sangon for sequencing.

### 4.3. Bioinformatics Analysis

Biology Online website ORFfinder (https://www.ncbi.nlm.nih.gov/orffinder/), TMHMM (https://services.healthtech.dtu.dk/service.php?TMHMM-2.0) and SignalIP (https://services.healthtech.dtu.dk/service.php?SignalP-4.1) were used to predict the molecular mass, theoretical pI, grand average of hydropathicity and signal peptide. The conserved domain of FaWRKY71 was predicted by the online website Conserved domains (https://www.ncbi.nlm.nih.gov/Structure/cdd/wrpsb.cgi). The phylogenetic tree was constructed using MEGA 6 software according to the obtained amino acid sequence of FaWRKY71. Using the PlantCARE website (http://bioinformatics.psb.ugent.be/webtools/plantcare/html/) to analyze the cis-acting elements of promoter FaWRKY71.

### 4.4. qRT-PCR

RNA from each sample was extracted and cDNA generated (as described above). Beacon Designer 8 software (Version: 8.14, USA) was used to design qRT-PCR primers. The 18S-26S interspacer RNA was used as the internal reference gene [51], all primers used are listed in Supplementary Data Table S1, and the relative expression was calculated using the 2^−ΔΔCt^ method [52]. Three biological replicates were set, and each repeats three times.

### 4.5. Subcellular Localization of FaWRKY71 in Nicotiana Benthamiana

The subcellular localization vector PMDC-EYFP was digested by *BamHI* and *EcoRI*, then CDS of *FaWRKY71* was fused to the N-terminal of EYFP protein by homologous recombination technique. m-Cherry was used as a nuclear localization Marker.. The fusion construct (FaWRKY71-EYFP) and a nuclear localization marker m-Cherry were transfected into Agrobacterium tumefaciens strain GV3101. Then infected tobacco leaves, and the epidemic cells were observed using a ZEISS 800 confocal microscope (Carl Zeiss, Germany).

### 4.6. Transient Overexpression in Strawberry Fruits

The CDS of *FaWRKY71* were combined into the pCAMBIA1301 vector with CaMV35s promoter and transferred into the ‘Xiaobai’ fruit (White stage) via the agrobacterium-mediated transformation method [53]. The agrobacterium GV3101 strain was cultured overnight in YEB medium containing 10 mM MES (pH 5.6) and appropriate antibiotics at 28 °C until the OD_600_ reach 1.0, centrifuge and remove the supernatant, then re-suspend the bacteria and adjust the OD_600_ to 0.8. After the agrobacterium suspension was incubated in MMA medium for 1 h, injected 400–600 µL of bacterial solution into the fruits, and fruits injected with an empty vector were used as control. Harvest when the fruit turns all red. Select at least 3 plants with 3 fruits per plant.

### 4.7. Determination of ROS Scavenging-Related Enzyme Activity, MDA Content, and Anthocyanin Content

The enzyme activity of SOD, POD, and CAT as well as the content of MDA and anthocyanin were measured using a Thermo Scientific Microplate Reader. Crude enzyme extract: 0.5 g fruit sample was homogenized in 5 mL phosphate buffer (pH 7.8, 50 mM) with a chilled mortar, and then centrifuged at 10,000 g for 20 min (4 °C), collect and measure the total volume (V) of the supernatant (Crude enzyme extract), and stored at 0–4 °C for later use.

SOD was assayed using the method of Robert et al. (1980) [54] with modifications. (1) Reaction mixture: phosphate buffer (pH 7.8, 50 mM): 130 mM methionine: 750 µM nitro blue tetrazolium: 20 µM riboflavin: 100 μM EDTA-Na_2_: H_2_O = 15:3:3:3:3:2.5; (2) Determination of SOD: The test tubes numbered 1, 2 and 3 were added 3 mL reaction mixture, then add 40 µL crude enzyme extract to tube 1, 40 µL phosphate buffer to tube 2 and 3 instead. Expose to light at 4,000 Lux for 30 min, absorbance at 560 nm was measured. The volume of the enzyme that inhibits 50% nitro blue tetrazolium photoreduction was defined as one unit of SOD activity (U).

POD activity was determined according to Yang et al. (2009) [55] with modifications. (1) Reaction mixture: consisted of 50 mL phosphate buffer (pH 6.0, 100 mM), 28 µL guaiacol, and 19 µL 30% H_2_O_2_; (2) Determination of POD: 8 µL crude enzyme extract and 300 µL reaction mixture were added to a microplate well, absorbance increments at 470 nm were recorded at 30 s intervals. One unit of POD activity (U) was defined as an increase in absorbance of 0.01 per minute per gram of sample.

CAT activity was measured as described by Yang et al. (2009) [55] with minor changes. (1) Reaction mixture: 0.1 M H_2_O_2_: 0.1 M phosphate buffer (pH 7.0) = 1:4; (2) Determination of CAT: 5.8 μL crude enzyme extract and 290 μL reaction mixture were added to a quartz glass UV microplate plate, absorbance was measured at 30 s intervals at 240 nm. One unit of CAT activity (U) was taken as the amount of enzyme with absorbance reduced by 0.1 per minute per gram of sample.

MDA content was performed as described by Wang et al. (2005) [56] with slight modifications: (1) 0.6 g thiobarbituric acid in a constant volume of 100 mL 10% trichloroacetic acid; (2) Determination of MDA: 1 mL crude enzyme extract and 2 mL reaction mixture were mixed then heated at 95 °C for 20 min, quickly cooled and centrifuged, the absorbance of the supernatant was measured at 450 nm, 523 nm, and 600 nm, respectively. The unit of MDA was expressed as μmol g^−1^ FW.

According to the method of Giusti et al. (2001) [57], the content of anthocyanin was determined. The absorbance was measured at 496 nm and 700 nm, respectively. The unit of anthocyanin (measured as pelargonidin 3-glucoside) was expressed as mg/g.

### 4.8. Section Staining and Microstructure Observation

The fruit was sliced into 2–3 mm pieces and placed in 30% alcohol for 5 min then transferred in water; Stained with 0.1% saffron for 12–24 h; Removed the saffron solution, rinsed several times with water, and put back in 50% alcohol for 5 min; Transferred into 70% alcohol and decolorized until lignified cell walls appear red; Sections were observed and photographed using an Inverted Microscope (Phenix, PH-XDS5).

### 4.9. Statistical Analysis

The experimental data were analyzed by SPSS Statistics Software, version 23. Error bars are SEs for three replicates, and statistical significance was determined by Student’s t-test (**, *p* < 0.01; *, *p* < 0.05). Multiple comparisons were tested using Turkey’s test and significant differences (*p* < 0.05) are indicated by different lowercase letters.

## 5. Conclusions

WRKY TFs play a momentous regulatory role in plant growth and development. *FaWRKY71* responds to abiotic stresses such as ABA, drought, salt, and cold. Apart from promoting fruit coloring by increasing anthocyanin content, *FaWRKY71* also facilitates fruit ripening and softening fruit texture by modulating the expression of *FaARF6/8* and *FaPG* as well as maintaining fruit cell morphology by increasing cell wall lignin content. In addition, the antioxidant capacity of strawberry fruit has also increased (Figure 5c). This study helps to reveal the response pattern of WRKY to stresses and provides a new perspective to regulate anthocyanin accumulation and fruit ripening in strawberry fruit.

## Figures and Tables

**Table 1:** The important cis-acting regulatory elements in the promoter sequence of *FaWRKY71*.

Cis-Acting Element	Function of Site	Sequence	Number
ABRE	cis-acting element involved in the abscisic acid responsiveness	CACGTG	1
AE-box	part of a module for light response	AGAAACAA	1
ARE	cis-acting regulatory element essential for the anaerobic induction	AAACCA	5
Box 4	part of a conserved DNA module involved in light responsiveness	ATTAAT	1
CGTCA-motif	cis-acting regulatory element involved in the MeJA-responsiveness	CGTCA	3
G-Box	cis-acting regulatory element involved in light responsiveness	CACGTG	1
MBS	MYB binding site involved in drought-inducibility	CAACTG	1
MRE	MYB binding site involved in light responsiveness	AACCTAA	1
MYB	MYB binding site	CAACCA/ TAACTG	1/1
MYC	MYB binding site	CATTTG/CATGTG	2/1
Sp1	light responsive element	GGGCGG	1
TGA-box	part of an auxin-responsive element	TGACGTAA	1

## Data Availability

Not applicable.

## Supplementary Materials

The following supporting information can be downloaded at: https://www.mdpi.com/article/10.3390/ijms232012483/s1, Table S1: The primers used in this study.

## References

[1] Eulgem T., Rushton P.J., Robatzek S., Somssich I.E. (2000). The WRKY superfamily of plant transcription factors. Trends Plant Sci..

[2] Rushton P.J., Somssich I.E., Ringler P., Shen Q.J. (2010). WRKY transcription factors. Trends Plant Sci..

[3] Viana V.E., Busanello C., da Maia L.C., Pegoraro C., Costa de Oliveira A. (2018). Activation of rice WRKY transcription factors: An army of stress fighting soldiers?. Curr. Opin. Plant Biol..

[4] Ishiguro S., Nakamura K. (1994). Characterization of a cDNA encoding a novel DNA.binding protein, SPF1, that recognizes SP8 sequences in the 5′ upstream regions of genes coding for sporamin and p.amylase from sweet potato. Mol. Gen. Genet..

[5] Wang M., Vannozzi A., Wang G., Liang Y.H., Tornielli G.B., Zenoni S., Cavallini E., Pezzotti M., Cheng Z.M. (2014). Genome and transcriptome analysis of the grapevine (*Vitis vinifera* L.) WRKY gene family. Hortic. Res..

[6] Meng D., Li Y.Y., Bai Y., Li M.J., Cheng L.L. (2016). Genome-wide identification and characterization of WRKY transcriptional factor family in apple and analysis of their responses to waterlogging and drought stress. Plant Physiol. Biochem..

[7] Jiang J.J., Ma S.H., Ye N.H., Jiang M., Cao J.S., Zhang J.H. (2017). WRKY transcription factors in plant responses to stresses. J. Integr. Plant Biol..

[8] Wei W., Liang D.W., Bian X.H., Shen M., Xiao J.H., Zhang W.K., Ma B., Lin Q., Lv J., Chen X. (2019). *GmWRKY54* improves drought tolerance through activating genes in abscisic acid and Ca^2+^ signaling pathways in transgenic soybean. Plant J..

[9] Zhu H., Jiang Y.N., Guo Y., Huang J.B., Zhou M.H., Tang Y.Y., Sui J.M., Wang J.S., Qiao L.X. (2021). A novel salt inducible WRKY transcription factor gene, *AhWRKY75*, confers salt tolerance in transgenic peanut. Plant Physiol. Biochem..

[10] Luo D.L., Ba L.J., Shan W., Kuang J.F., Lu W.J., Chen J.Y. (2017). Involvement of WRKY Transcription Factors in ABA-Induced Cold Tolerance of Banana Fruit. J. Agric. Food Chem..

[11] Wang Y.H., Zhao F.X., Zhang G., Jia S.Z., Yan Z.M. (2021). FaWRKY11 transcription factor positively regulates resistance to *Botrytis cinerea* in strawberry fruit. Sci. Hortic..

[12] Lei Y.Y., Sun Y.P., Wang B.T., Yu S., Dai H.Y., Li H., Zhang Z.H., Zhang J.X. (2020). Woodland strawberry WRKY71 acts as a promoter of flowering via a transcriptional regulatory cascade. Hortic Res..

[13] An J.P., Zhang X.W., You C.X., Bi S.Q., Wang X.F., Hao Y.J. (2019). MdWRKY40 promotes wounding-induced anthocyanin biosynthesis in association with MdMYB1 and undergoes MdBT2-mediated degradation. New Phytol..

[14] Li C., Wu J., Hu K.D., Wei S.W., Sun H.Y., Hu L.Y., Han Z., Yao G.F., Zhang H. (2020). PyWRKY26 and PybHLH3 cotargeted the PyMYB114 promoter to regulate anthocyanin biosynthesis and transport in red-skinned pears. Hortic. Res..

[15] Lin-Wang K., McGhie T.K., Wang M., Liu Y., Warren B., Storey R., Espley R.V., Allan A.C. (2014). Engineering the anthocyanin regulatory complex of strawberry (*Fragaria vesca*). Front. Plant Sci..

[16] Zhang J.J., Wang X., Yu O., Tang J.J., Gu X.G., Wan X.C., Fang C.B. (2011). Metabolic profiling of strawberry (*Fragaria* × *ananassa* Duch.) during fruit development and maturation. J. Exp. Bot..

[17] Medina-Puche L., Blanco-Portales R., Molina-Hidalgo F.J., Cumplido-Laso G., García-Caparrós N., Moyano-Cañete E., Caballero-Repullo J.L., Muñoz-Blanco J., Rodríguez-Franco A. (2016). Extensive transcriptomic studies on the roles played by abscisic acid and auxins in the development and ripening of strawberry fruits. Funct. Integr. Genomics.

[18] Symons G.M., Chua Y.J., Ross J.J., Quittenden L.J., Davies N.W., Reid J.B. (2012). Hormonal changes during non-climacteric ripening in strawberry. J. Exp. Bot..

[19] Li T.Y., Dai Z.R., Zeng B.Z., Li J., Ouyang J.Y., Kang L., Wang W., Jia W.S. (2022). Autocatalytic biosynthesis of abscisic acid and its synergistic action with auxin to regulate strawberry fruit ripening. Hortic. Res..

[20] Jiang Y.M., Joyce D.C. (2003). ABA effects on ethylene production, PAL activity, anthocyanin and phenolic contents of strawberry fruit. Plant Growth Regul..

[21] Moya-León M.A., Mattus-Araya E., Herrera R. (2019). Molecular Events Occurring During Softening of Strawberry Fruit. Front. Plant Sci..

[22] Paniagua C., Ric-Varas P., García-Gago J.A., López-Casado G., Blanco-Portales R., Muñoz-Blanco J., Schückel J., Knox J.P., Matas A.J., Quesada M.A. (2020). Elucidating the role of polygalacturonase genes in strawberry fruit softening. J. Exp. Bot..

[23] Posé S., Paniagua C., Cifuentes M., Blanco-Portales R., Quesada M.A., Mercado J.A. (2013). Insights into the effects of *polygalacturonase FaPG1* gene silencing on pectin matrix disassembly, enhanced tissue integrity, and firmness in ripe strawberry fruits. J. Exp. Bot..

[24] Wei W., Hu Y., Han Y.T., Zhang K., Zhao F.L., Feng J.Y. (2016). The WRKY transcription factors in the diploid woodland strawberry *Fragaria vesca*: Identification and expression analysis under biotic and abiotic stresses. Plant Physiol. Biochem..

[25] Chen P., Liu Q.Z. (2019). Genome-wide characterization of the *WRKY* gene family in cultivated strawberry (*Fragaria × ananassa* Duch.) and the importance of several group III members in continuous cropping. Sci. Rep..

[26] Zou X.H., Dong C., Liu H.T., Gao Q.H. (2022). Genome-wide characterization and expression analysis of WRKY family genes during development and resistance to Colletotrichum fructicola in cultivated strawberry (*Fragaria* × *ananassa* Duch.). J. Integr. Agr..

[27] Zhang Y.T., Jiang L.Y., Li Y.L., Chen Q., Ye Y.T., Zhang Y., Luo Y., Sun B., Wang X.R., Tang H.R. (2018). Effect of Red and Blue Light on Anthocyanin Accumulation and Differential Gene Expression in Strawberry (*Fragaria* × *ananassa*). Molecules.

[28] Wang L., Luo Z.S., Yang M.Y., Liang Z., Qi M., Dong Y.Y., Xu Y.Q., Lin X.Y., Li L. (2022). The action of RED light: Specific elevation of pelargonidin-based anthocyanin through ABA-related pathway in strawberry. Postharvest Biol. Technol..

[29] Malekzadeh Shamsabad M.R., Esmaeilizadeh M., Roosta H.R., Dąbrowski P., Telesiński A., Kalaji H.M. (2022). Supplemental light application can improve the growth and development of strawberry plants under salinity and alkalinity stress conditions. Sci. Rep..

[30] Zhang Y.T., Hu W.J., Peng X.R., Sun B., Wang X.R., Tang H.R. (2018). Characterization of anthocyanin and proanthocyanidin biosynthesis in two strawberry genotypes during fruit development in response to different light qualities. J. Photochem. Photobiol. B.

[31] Fei J., Wang Y.S., Cheng H., Su Y.B., Zhong Y.J., Zheng L. (2022). The Kandelia obovata transcription factor KoWRKY40 enhances cold tolerance in transgenic Arabidopsis. BMC Plant Biol..

[32] Zhang Y., Yu H.J., Yang X.R., Li Q., Ling J., Wang H., Gu X.F., Huang S.W., Jiang W.J. (2016). CsWRKY46, a WRKY transcription factor from cucumber, confers cold resistance in transgenic-plant by regulating a set of cold-stress responsive genes in an ABA-dependent manner. Plant Physiol. Biochem..

[33] Misson J., Raghothama K.G., Jain A., Jouhet J., Block M.A., Bligny R., Ortet P., Creff A., Somerville S., Rolland N. (2005). A genome-wide transcriptional analysis using *Arabidopsis thaliana* Affymetrix gene chips determined plant responses to phosphate deprivation. PNAS.

[34] Devaiah B.N., Karthikeyan A.S., Raghothama K.G. (2007). WRKY75 transcription factor is a modulator of phosphate acquisition and root development in Arabidopsis. Plant Physiol..

[35] Jaakola L. (2013). New insights into the regulation of anthocyanin biosynthesis in fruits. Trends Plant Sci..

[36] Zhang J.K., Wang Y.C., Mao Z.L., Liu W.N., Ding L.C., Zhang X.N., Yang Y.W., Wu S.Q., Chen X.S., Wang Y.L. (2022). Transcription factor McWRKY71 induced by ozone stress regulates anthocyanin and proanthocyanidin biosynthesis in Malus crabapple. Ecotoxicol. Environ. Saf..

[37] Schaart J.G., Dubos C., Romero De La Fuente I., van Houwelingen A.M.M.L., de Vos R.C.H., Jonker H.H., Xu W., Routaboul J.M., Lepiniec L., Bovy A.G. (2013). Identification and characterization of MYB-bHLH-WD40 regulatory complexes controlling proanthocyanidin biosynthesis in strawberry (*Fragaria* × *ananassa*) fruits. New Phytol..

[38] Wang H., Zhang H., Yang Y., Li M., Zhang Y.T., Liu J.S., Dong J., Li J., Butelli E., Xue Z. (2019). The control of red colour by a family of MYB transcription factors in octoploid strawberry (*Fragaria* × *ananassa*) fruits. Plant Biotechnol. J..

[39] An X.H., Tian Y., Chen K.Q., Liu X.J., Liu D.D., Xie X.B., Cheng C.G., Cong P.H., Hao Y.J. (2015). MdMYB9 and MdMYB11 are involved in the regulation of the JA-induced biosynthesis of anthocyanin and proanthocyanidin in apples. Plant Cell Physiol..

[40] Carlos R.F., Hernán G.R., Pedro M.C., Gustavo A.M., Raúl H., María A.M. (2010). Changes in cell wall polysaccharides and cell wall degrading enzymes during ripening of *Fragaria chiloensis* and *Fragaria* × *ananassa* fruits. Sci. Hortic..

[41] Zhang W.W., Zhao S.Q., Zhang L.C., Xing Y., Jia W.S. (2020). Changes in the cell wall during fruit development and ripening in *Fragaria vesca*. Plant Physiol. Biochem..

[42] Castro R.I., Muñoz-Vera M., Parra-Palma C., Valenzuela-Riffo F., Figueroa C.R., Morales-Quintana L. (2021). Characterization of cell wall modification through thermogravimetric analysis during ripening of Chilean strawberry (*Fragaria chiloensis*) fruit. Cellulose.

[43] Given N.K., Venis M.A., Gierson D. (1988). Hormonal regulation of ripening in the strawberry, a non-climacteric fruit. Planta.

[44] Li B.J., Grierson D., Shi Y.N., Chen K.S. (2022). Roles of abscisic acid in regulating ripening and quality of strawberry, a model non-climacteric fruit. Hortic. Res..

[45] Xu X.B., Ma X.Y., Le H.H., Yin L.L., Shi X.Q., Song H.M. (2015). MicroRNAs play an important role in the regulation of strawberry fruit senescence in low temperature. Postharvest Biol. Technol..

[46] Nagpal P., Ellis C.M., Weber H., Ploense S.E., Barkawi L.S., Guilfoyle T.J., Hagen G., Alonso J.M., Cohen J.D., Farmer E.E. (2005). Auxin response factors ARF6 and ARF8 promote jasmonic acid production and flower maturation. Development.

[47] Apel K., Hirt H. (2004). Reactive oxygen species: Metabolism, oxidative stress, and signal transduction. Annu. Rev. Plant Biol..

[48] Shen N., Wang T.F., Gan Q., Liu S., Wang L., Jin B. (2022). Plant flavonoids: Classification, distribution, biosynthesis, and antioxidant activity. Food Chem..

[49] Chen Q., Yu H.W., Wang X.R., Xie X.L., Yue X.Y., Tang H.R. (2012). An alternative cetyltrimethylammonium bromide-based protocol for RNA isolation from blackberry (*Rubus* L.). Genet. Mol. Res..

[50] Jiang L.Y., Yue M.L., Liu Y.Q., Ye Y.Y., Zhang Y.T., Lin Y.X., Wang X.R., Chen Q., Tang H.R. (2022). Alterations of Phenylpropanoid Biosynthesis Lead to the Natural Formation of Pinkish-Skinned and White-Fleshed Strawberry (*Fragaria* × *ananassa*). Int. J. Mol. Sci..

[51] Raab T., López-Ráez J.A., Klein D., Caballero J.L., Moyano E., Schwab W., Muñoz-Blanco J. (2006). FaQR, required for the biosynthesis of the strawberry flavor compound 4-hydroxy-2,5-dimethyl-3(2H)-furanone, encodes an enone oxidoreductase. Plant Cell..

[52] Livak K.J., Schmittgen T.D. (2001). Analysis of relative gene expression data using real-time quantitative PCR and the 2^−∆∆CT^ Method. Methods.

[53] Lin Y.X., Jiang L.Y., Chen Q., Li Y.L., Zhang Y.T., Luo Y., Zhang Y., Sun B., Wang X.R., Tang H.R. (2018). Comparative Transcriptome Profiling Analysis of Red- and White-Fleshed Strawberry *(Fragaria × ananassa*) Provides New Insight into the Regulation of the Anthocyanin Pathway. Plant Cell Physiol..

[54] Stewart R.R., Bewley J.D. (1980). Lipid Peroxidation Associated with Accelerated Aging of Soybean Axes. Plant Physiol..

[55] Yang Z.F., Zheng Y.H., Cao S.F. (2009). Effect of High Oxygen Atmosphere Storage on Quality, Antioxidant Enzymes, and DPPH-Radical Scavenging Activity of Chinese Bayberry Fruit. J. Agric. Food Chem..

[56] Wang Y.S., Tian S.P., Xu Y. (2005). Effects of high oxygen concentration on pro- and anti-oxidant enzymes in peach fruits during postharvest periods. Food Chem..

[57] Giusti M.M., Wrolstad R.E. (2001). Characterization and Measurement of Anthocyanins by UV-Visible Spectroscopy. Current Protocols in Food Analytical Chemistry.

